# Limits of Kirchhoff’s Laws in Plasmonics

**DOI:** 10.1038/s41598-018-20239-x

**Published:** 2018-01-30

**Authors:** Gary Razinskas, Paolo Biagioni, Bert Hecht

**Affiliations:** 1 Nano-Optics and Biophotonics Group, Department of Experimental Physics 5, University of Würzburg, Am Hubland, D-97074 Würzburg, Germany; 2 Physics Department, Politecnico di Milano, Piazza Leonardo da Vinci 32, I-20133 Milano, Italy; 3 Röntgen Center for Complex Material Systems (RCCM), Am Hubland, D-97074 Würzburg, Germany

## Abstract

The validity of Kirchhoff’s laws in plasmonic nanocircuitry is investigated by studying a junction of plasmonic two-wire transmission lines. We find that Kirchhoff’s laws are valid for sufficiently small values of a phenomenological parameter *κ* relating the geometrical parameters of the transmission line with the effective wavelength of the guided mode. Beyond such regime, for large values of the phenomenological parameter, increasing deviations occur and the equivalent impedance description (Kirchhoff’s laws) can only provide rough, but nevertheless useful, guidelines for the design of more complex plasmonic circuitry. As an example we investigate a system composed of a two-wire transmission line and a nanoantenna as the load. By addition of a parallel stub designed according to Kirchhoff’s laws we achieve maximum signal transfer to the nanoantenna.

## Introduction

The strong subwavelength light confinement of guided plasmonic modes supported by noble-metal nanowires^1–3^ is a prerequisite for the realization of optical nanocircuits bridging the size mismatch between nanoelectronics and micrometer-scaled optical devices. At the core of such optical nanocircuits lies a very small footprint network of optical transmission lines enabling the controlled distribution and manipulation of plasmonic excitations. Devices featuring well-defined built-in functionalities^4,5^ such as directional switching^6–8^ or filtering^9^ have been realized. Junctions of such transmission lines represent one of the fundamental building blocks of any such network.

The power of electronic circuit design is based on the fact that complex circuitry can be replaced by simple circuit elements described by a discrete impedance. Circuit elements can then be arranged into networks whose functionality can be analyzed based on Kirchhoff’s laws. Naturally, some efforts have also been devoted to the use of Kirchhoff’s laws for the description of circuit elements and circuitry at optical frequencies. The figurative representation and modularization of nano-optical systems enabled by the application of equivalent circuit models provides significant insight into their optical response and can be used to improve the overall performance of plasmonic nanostructures such as isolated nanospheres^10^, dipole^11–16^ and more elaborate^17^ nanoantennas, or plasmonic waveguide components^18,19^. As a well-established principle of classical transmission line theory, the impedance description of lumped and distributed circuit elements^13,20,21^ has also been successfully applied to improve the impedance matching between a plasmonic waveguide and a nanoantenna^18,22^ and between waveguide segments of different geometry and/or orientation^23–26^. However, at the short wavelengths involved and given the finite dimensions of nano-optical circuitry the applicability of Kirchhoff’s laws cannot be taken for granted. Yet there is no systematic study as to what extent circuit theory can be applied to subwavelength plasmonic systems.

In spite of the similarities, the optical (plasmonic) regime shows significant differences from the radio frequency (RF) regime. Firstly, at optical frequencies noble metals do not behave as perfect conductors but, due to the small negative real part of their permittivity, as plasmonic materials. This holds several implications, for example the non-negligible skin depth compared to the wire cross section, which in turn creates volume currents with no counterparts in RF^27^. Secondly, the lumped element model of electronic circuits and Kirchhoff’s laws can be understood as an approximation of Maxwell’s equations in the low-frequency domain (quasistatic limit), equivalent to assuming the involved wires, when compared to the circuit’s operation wavelength, as quasi one-dimensional in cross section and their junctions as point-like objects. While in the RF regime with typical ratios of wire dimension to wavelength on the order of 10^−3^ (estimated for a common twin lead cable at 100 MHz frequency) this limit is satisfied, in the optical regime the approximation has to be questioned. Even with state-of-the-art fabrication techniques the realizable dimensions (i.e. the nanowire cross-section and the inter-wire distance) are in the range of a few tens of nanometers, thus such realistic nanowires and their junctions are rather laterally extended objects in a size range comparable to the effective wavelength of the supported eigenmodes, which is reflected by ratios of wire dimension to wavelength exceeding 0.1, as further detailed below. Finally, at variance with the purely transverse guided modes sustained by perfect conductors (which is the case in the RF regime), plasmonic modes possess a significant longitudinal component that is not taken into account in the standard impedance description.

Here, we investigate the validity of the Kirchhoff’s circuit laws in the optical regime by considering a fundamental, yet simple system, i.e. a junction of two-wire transmission lines (TWTLs) supporting an antisymmetric guided plasmonic mode. The first part of this work deals with an idealized junction of infinite TWTLs of uniform cross sectional geometry with one input and two outputs. We show by finite-difference time-domain (FDTD) simulations that clear deviations occur from the expected behavior derived from Kirchhoff’s circuit laws applied to the same waveguide junction. By varying the TWTL’s cross sectional dimensions, the deviation from Kirchhoff theory can clearly be correlated with an increasing finite extension of the structure. Despite the significant deviations, which cannot be completely neglected even for nanowire dimensions at the limit of current microfabrication techniques, we show that Kirchhoff’s laws can still be used as a qualitative guideline to compose nano-optical circuitry that is then subject to further (numerical) optimization. The second part of this work highlights additional deviations due to mutual coupling of discrete circuit components located in close proximity based on the example of a system composed of a TWTL and a nanoantenna as the load. By addition of a parallel stub designed according to Kirchhoff’s laws we realize maximum transfer of signal between circuit elements, a necessary prerequisite for the design of efficient devices, and use this example to further test the validity of the lumped-element impedance approach.

## Materials and Methods

**Figure 1:**
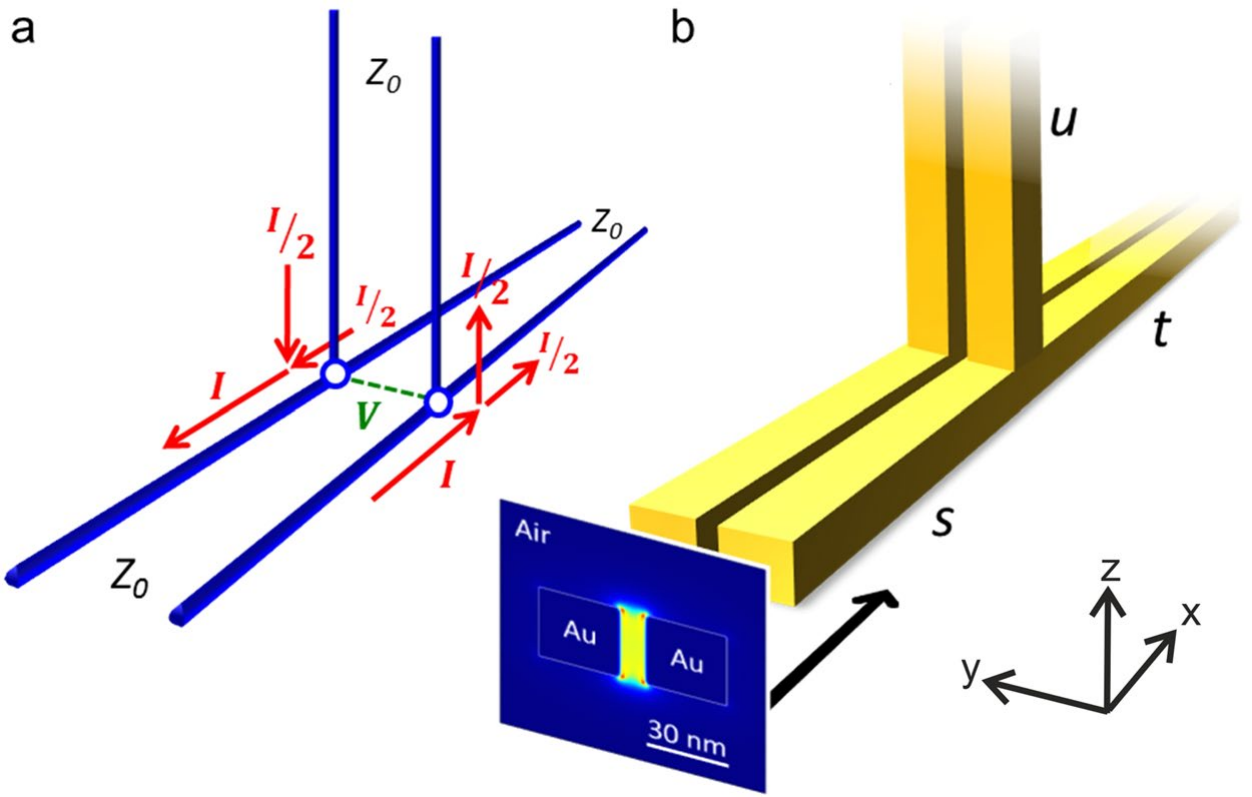
Classical vs. plasmonic transmission lines. (**a**) Two classical TWTLs of identical characteristic impedance *Z*_0_ connected in parallel resulting in an equal splitting of electrical currents at the point junction. (**b**) Equivalent junction of two plasmonic TWTLs, an idealized building block of optical nanocircuits. (inset) The guided antisymmetric mode (electric field intensity |*E*|^2^ at free-space wavelength *λ* = 830 nm) is directly launched from the left and propagates along the nano-sized TWTL, where the mode intensity is split at the parallel junction. The mode symmetry is defined by the symmetry of the longitudinal field component *E*_*x*_ with respect to the TWTL mid-plane.

A classical TWTL consists of a pair of parallel conducting wires separated by a uniform distance. Wire geometry and separation determine its characteristic impedance *Z*_0_. It is a fact of classical transmission line theory that the parallel connection of two such idealized infinitely long TWTLs (as shown in Fig. 1a) results in an equal splitting of any input current. This is an implication of the node analysis by means of Kirchhoff’s circuit laws. To be more specific, if two impedances are connected in parallel, the voltage drop across both of them is identical, thus according to Ohm’s law the current entering the junction is split inversely proportional to their impedances. Therefore, for two identical parallel impedances the current splits equally. The optical analogue, the simplified plasmonic TWTL junction geometry studied in the following, is sketched in Fig. 1b. An input TWTL (marked as *s* in Fig. 1b) of characteristic impedance *Z*_0_ branches into two perpendicularly oriented TWTLs (marked as *t* and *u* in Fig. 1b) of identical cross section extending to infinity. For reasons of simplicity the investigated TWTL structure is placed in vacuum to ensure that both the vertical and the horizontal TWTLs support the same propagating mode with identical *Z*_0_ and to exclude any parasitic effects of a substrate. In the simulations presented here, two nanowires of squared cross section consisting of gold and separated by a small gap build up the TWTL. The dielectric function of gold is modeled according to experimental data^28^ fitted with an analytical model^29^. In a first step, we calculate the mode profile of the antisymmetric TWTL eigenmode at a wavelength *λ* = 830 nm (Fig. 1b, inset) for a given wire cross section by means of a full-vectorial eigenmode solver (MODE Solutions, Lumerical Solutions Inc.). Afterwards, full-3D FDTD simulations (FDTD Solutions, Lumerical Solutions Inc.) are carried out at the same wavelength exciting the structure from the input TWTL side (*s*) by directly injecting the previously calculated mode. Infinitely long TWTLs are mimicked in the simulations by extending them into perfectly matched layer boundaries, thus avoiding any back-reflection to the junction region. The whole TWTL junction region is covered by a uniform mesh of 1 × 1 × 1 nm^3^ cell size.

## Results and Discussion

**Figure 2:**
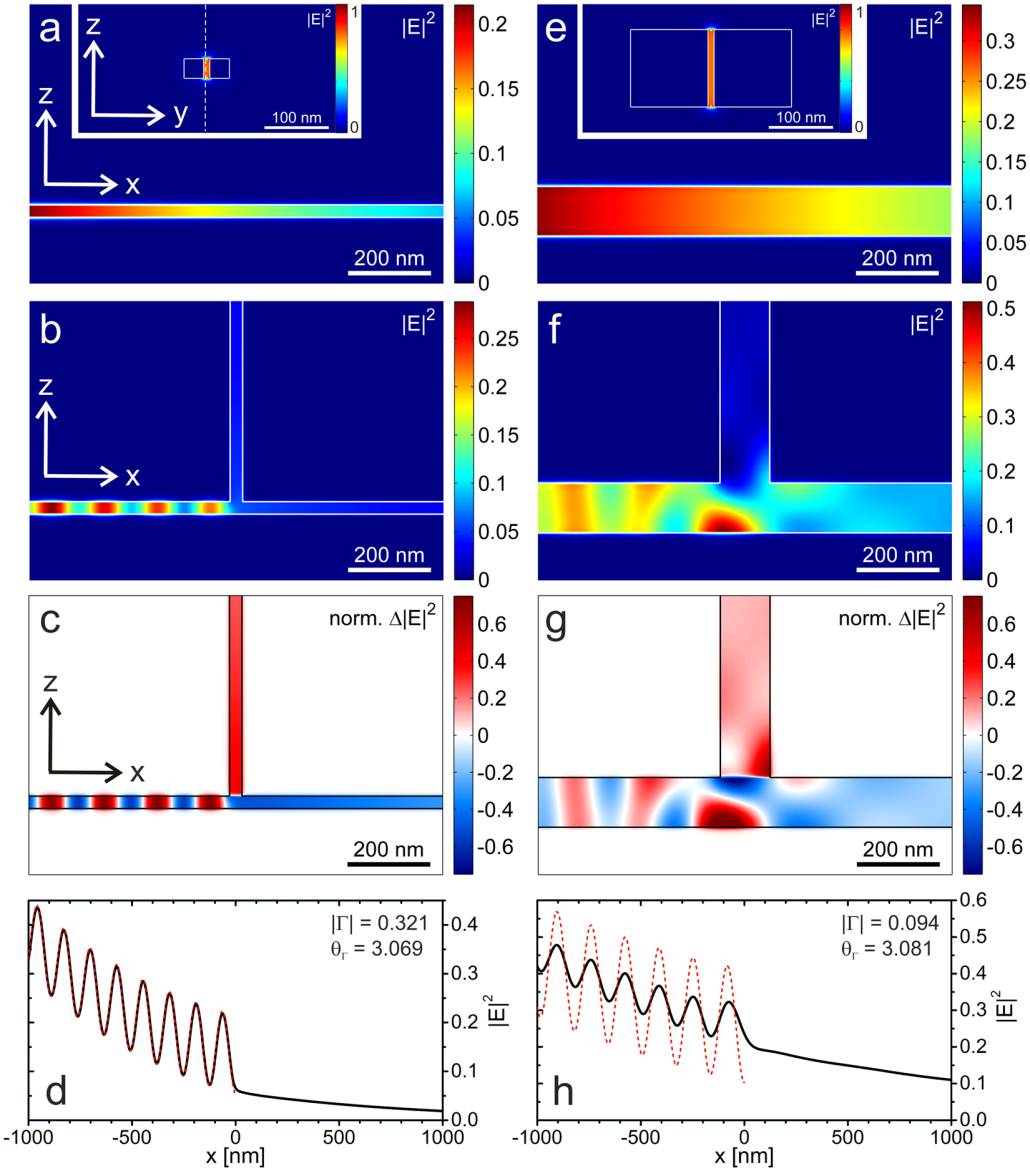
Parallel junction of plasmonic TWTLs. (**a**) Optical near-field intensity distribution in a cut through the gap centered between small-sized nanowires (width = height = 30 nm, gap = 10 nm) of an unconnected TWTL. Inset: modal profile of the guided antisymmetric mode with most of its intensity localized in the nanowire gap. The dashed white line indicates the plane used to record the near-field intensity cut. (**b**) Same as in (**a**) but for a parallel junction of TWTLs. (**c**) Intensity difference plot obtained from the maps with (**b**) and without (**a**) the upward pointing semi-infinite TWTL. (**d**) Standing wave pattern forming along the TWTL due to partial reflection of the antisymmetric mode at the TWTL junction used to extract the reflection coefficient. The theoretically expected behavior (red dashed line) corresponding to |Γ| = 1/3 and *θ*_Γ_ = *π* is added as a guide for the eye. The intensity distribution (black solid line) is recorded mid-height in the gap center of the horizontal TWTL. (**e**–**h**) The same as (**a**–**d**), but for a parallel junction of wider TWTLs (width = height = 120 nm, gap = 10 nm).

Figure 2a–d show simulation results for a small-sized TWTL composed of nanowires with a 30 × 30 nm^2^ cross section separated by a 10 nm gap. When exciting the TWTL’s antisymmetric eigenmode (see Fig. 2a, inset) in a single infinite TWTL, the intensity is exponentially damped along the propagation direction (Fig. 2a). As the parallel junction is formed by introducing the upward TWTL (*u*) (Fig. 2b) the intensity transmitted through the junction region into the horizontal output TWTL (*t*) is significantly reduced, in favor of both mode intensity propagating in the upward direction and mode intensity being reflected due to the impedance mismatch at the junction. The latter results in the formation of a standing wave intensity pattern on the input side, as obvious from Fig. 2c, where we plot the intensity difference between the simulation results of Fig. 2b and those of Fig. 2a.

We introduce two figures of merit to analyze the junction and quantify the difference between the plasmonic TWTL junctions and their RF counterparts, namely the power splitting ratio and the (complex) reflection coefficient ${\rm{\Gamma }}=|{\rm{\Gamma }}|{e}^{j{\theta }_{{\rm{\Gamma }}}}$, where |Γ| and *θ*_Γ_ denote the reflection amplitude and phase, respectively, and *j* is the imaginary unit. Firstly, the power splitting ratio is obtained by applying a mode-overlap analysis using 2D near-field data in cuts through the horizontal (vertical) output TWTL at equal distances from the junction center to determine the remaining power in the antisymmetric mode in the horizontal (vertical) output. With this, the power splitting ratio is determined as the ratio of powers in the antisymmetric mode along the horizontal and the vertical output TWTL, respectively. As a measure for the deviations from the RF behavior we exploit the fact that in classical transmission line theory Kirchhoff’s circuit laws together with Ohm’s law predict an equal splitting of power at a parallel junction of two identical semi-infinite TWTLs.

Secondly, further insight into the behavior of the junction region is obtained by determining Γ at the junction position as a direct measure of the degree of impedance matching. The input impedance *Z*_tot_ of a parallel junction of horizontal and vertical semi-infinite output TWTLs with the same *Z*_0_ is *Z*_tot_ = *Z*_0_/2, which is not impedance matched to the characteristic impedance of the input TWTL. Consequently, the guided plasmon mode is partially reflected as it reaches the junction, which according to the definition of Γ,

1 $${\rm{\Gamma }}=\frac{{Z}_{tot}-{Z}_{0}}{{Z}_{tot}+{Z}_{0}},$$

results in a theoretically expected reflection amplitude |Γ| = 1/3 and a phase accumulation *θ*_Γ_ = *π*. In numerical simulations, Γ is obtained by fitting the total intensity *I*_total_ of the standing wave pattern building up along the input TWTL along a linecut centered in the TWTL gap with the following analytical model^18^,

2 $${I}_{total}(x)=|{A}_{0}\,[{e}^{jkx}+{e}^{jk(X-x)}\,{\rm{\Gamma }}{e}^{jkX}]{|}^{2},$$

where *k* and *A*_0_ denote wave vector and initial field amplitude of the mode, respectively. *k* is obtained from the eigenmode solver as the eigenvalue for the respective cross-sectional TWTL geometry. The geometrical junction center is set to position *X*.

For the geometry studied in Fig. 2a–d one observes that the intensity in the antisymmetric mode entering the horizontal output is only 10% larger than the intensity directed into the vertical output. Similarly, as shown in Fig. 2d, the reflection amplitude Γ = 0.321 and phase *θ*_Γ_ = 3.069 obtained by fitting of Eq. 2 to the standing wave pattern also reveal very small differences from transmission line theory predictions of 3.7% and 2.3%, respectively. In the regime investigated in Fig. 2a–d, therefore, Kirchhoff’s laws respresent a valuable asset, providing accurate predictions for the response of the plasmonic circuit.

**Figure 3:**
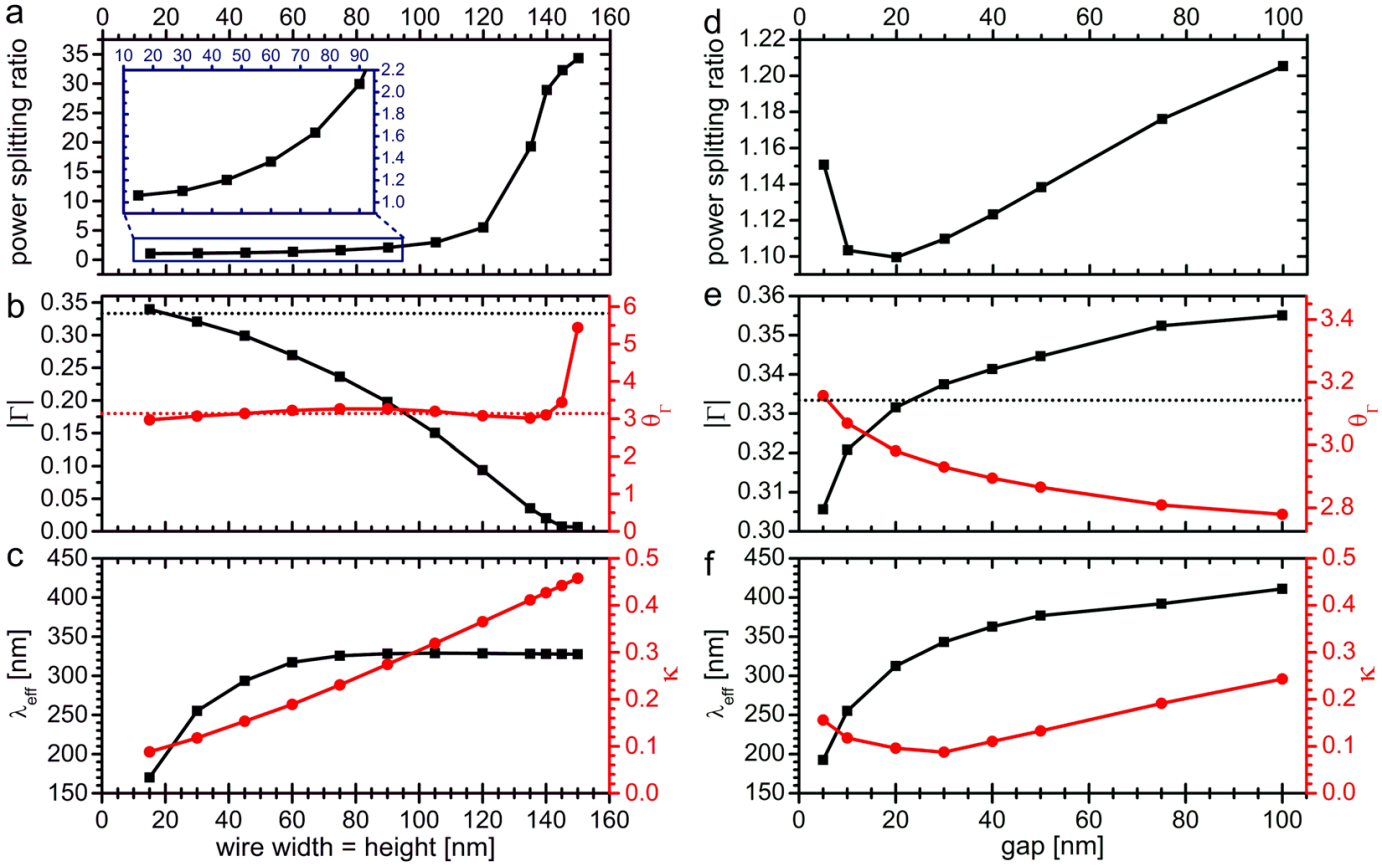
Variation of plasmonic TWTL dimensions. (**a**) Power splitting ratio, (**b**) reflection amplitude (black curve) and phase (red curve), and (**c**) *λ*_eff_ (black curve) and *κ* (red curve) as a function of nanowire width. The gap is kept constant at 10 nm. (**d**) Power splitting ratio, (**e**) reflection amplitude (black curve) and phase (red curve), and (**f**) *λ*_eff_ (black curve) and *κ* (red curve) as a function of the gap size. The nanowire width and the height are kept constant at 30 nm.

The influence of the structure size on the observed deviations from RF theory is demonstrated in Fig. 2e–h, showing equivalent simulation results for a wider TWTL with nanowire cross section of 120 × 120 nm^2^ and a gap of 10 nm. Here, the intensity distribution in the gap region becomes notably asymmetric, as seen in Fig. 2f,g, leading to the presence of anomalous field gradients at the junction, which deviate markedly from the expected transversal wavefronts of the guided modes. The resulting deviation from the RF-like behavior is manifested in a very poor power splitting ratio of 5.51 and calculated reflection amplitude Γ = 0.094 and phase *θ*_Γ_ = 3.081 (Fig. 2h). To investigate in more detail the influence of the lateral dimensions of the plasmonic waveguide on the degree of agreement with RF theory, we systematically varied the nanowire width (Fig. 3a–c) and the gap distance (Fig. 3d–f), separately.

On the one side, as the width and the height of the nanowires with square cross section are simultaneously increased (with a fixed gap of 10 nm) the power splitting ratio (Fig. 3a) gradually increases and deviates more from the expected ratio of 1. Thus, especially for wider wires, the guided mode tends to propagate mostly horizontally, whereas only little intensity is re-directed to the upward oriented TWTL. Similarly, the reflection amplitude displayed in Fig. 3b (black curve) drastically decreases for increasing wire dimensions, again deviating more and more from the expected value of |Γ| = 1/3. It is noted that the reflection phase (Fig. 3b, red curve) in contrast does not show such a monotone behavior, but still shows a strong deviation from the expected value of *θ*_Γ_ = *π* beyond a wire width of 140 nm. The observed increase in deviations from RF theory derived from the two figures of merit can be tentatively correlated to a phenomenological characteristic TWTL dimension *κ*, which is defined as the ratio of the largest TWTL physical dimension, which can be either the nanowire width or the gap distance, and the effective wavelength *λ*_eff_ of the antisymmetric mode supported by the specific TWTL cross section. Originally, the lumped circuit model and consequently Kirchhoff’s circuit laws were derived under the assumption of very small characteristic circuit cross sections compared to the circuit’s operation wavelength, i.e. $\kappa \ll 1$. Figure 3c shows the evolution of *λ*_eff_ (black curve) as a function of the nanowire width, demonstrating an initial increase of *λ*_eff_ followed by a saturation behavior for wires exceeding about 100 nm in width. With this the evolution of the phenomenological parameter *κ* is derived, showing a steady increase as the nanowire width grows and reaches values of almost 0.5 for the widest wires studied (Fig. 3c, red curve). Here, the validity of the assumption of $\kappa \ll 1$ clearly breaks down, an occurrence that correlates with the strongly deviating behavior of TWTL junctions composed of extended nanowires.

On the other side, as the gap is increased (with a fixed cross section of 30 × 30 nm^2^) the power splitting ratio (Fig. 3d) first decreases reaching a minimum (and thus best agreement with theory) for a gap of 20 nm, then it starts increasing again for larger gaps. However, even for the largest investigated gap of 100 nm the deviation remains rather small (less than 20% increase). Similarly, the reflection amplitude (Fig. 3e, black curve) shows best agreement with the expected value of |Γ| = 1/3 for a gap width of 20 nm. This behavior can be again phenomenologically correlated with the relative characteristic TWTL dimension *κ*. As shown in Fig. 3f, *λ*_eff_ increases with gap size, however the TWTL’s largest physical dimension is not changing up to a gap of 30 nm, since only then the gap distance and the nanowire width become equal in size. Therefore, *κ* is not monotonically increasing, but instead shows a minimum for 30 nm gap sizes. Also, in the presented gap scan *κ* has overall smaller values compared to the nanowire width scan (Fig. 3c), supporting the generally better agreement with the lumped circuit model.

**Figure 4:**
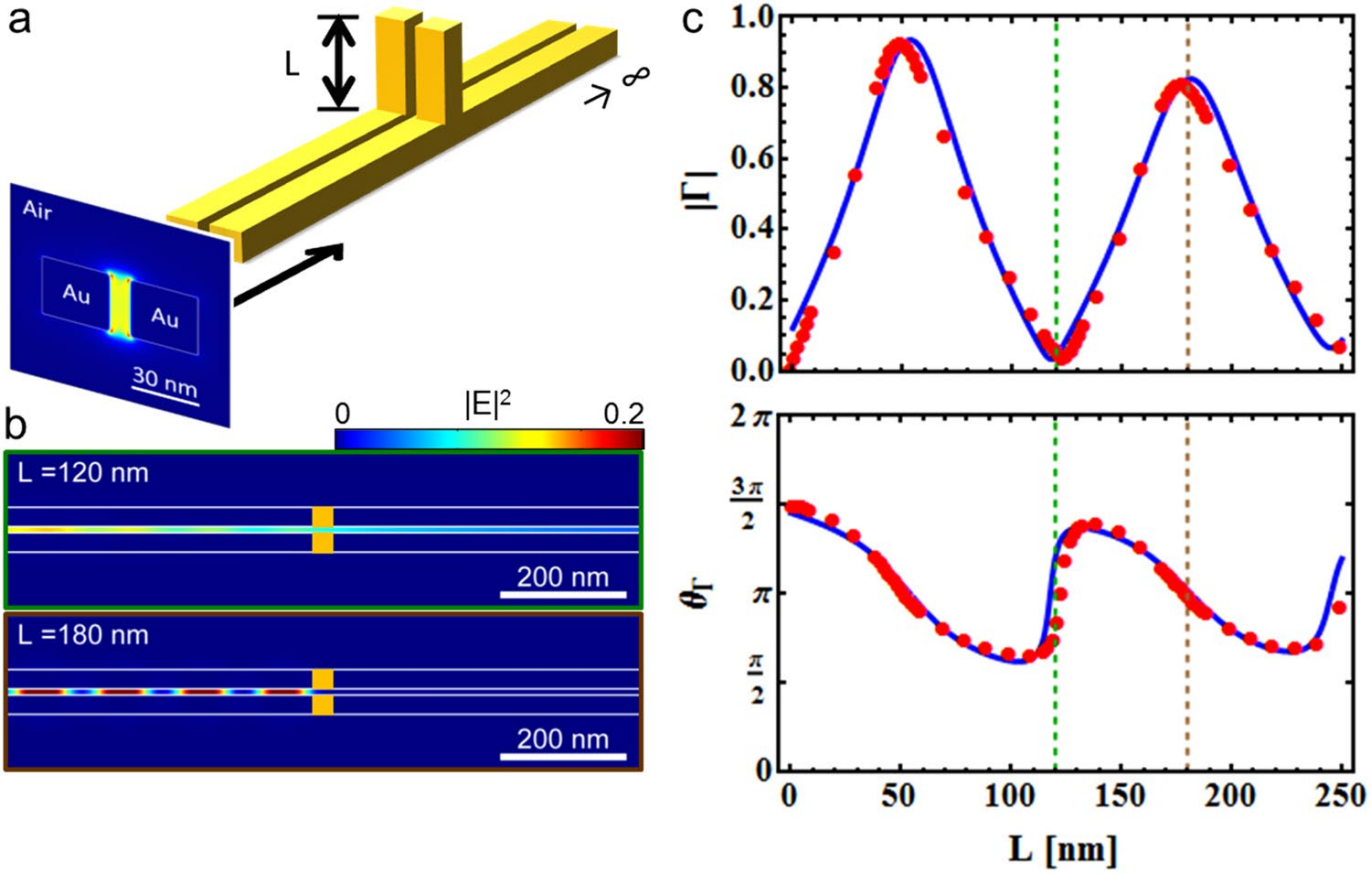
Finite stub reflectivity tuning. (**a**) Sketch of the investigated system featuring an infinite TWTL connected in parallel with a finite stub of length *L*. The shown antisymmetric mode is directly launched from the left and propagates along the nano-sized TWTL. (**b**) The mode’s standing wave pattern along a cut at midheight through the TWTL for high impedance stub (*L* = 120 nm, top) and low impedance stub (*L* = 180 nm, bottom). (**c**) Reflection amplitude (top) and phase (bottom) for systems of varying stub length *L*. The red dots are obtained by fitting of FDTD simulation data with the model described in Eq. 2, while the blue solid lines are obtained by the analytical model (Supporting Information).

Based on this analysis, and thanks to the improved understanding gained on the reasons and the relevance of the deviations from Kirchhoff’s laws, we can infer that it is possible with reasonable accuracy to extend the range of validity of Kirchhoff’s analysis to plasmonic waveguide networks in the optical regime provided that $\kappa \,\lessapprox \,0.1$. In this case the lumped circuit approximation commonly used in the RF regime can be applied on safe grounds. It should be noted, though, that the numerical case study at hand of course cannot be taken as a general proof for the validity of the *κ*-criterion. Nevertheless, it is expected that similar threshold values will be found in different systems as well. This is highly beneficial because it allows one to simplify complex relevant problems such as the optimization of power transfer in a system composed of a TWTL and a nanoantenna as the load by addition of a suitable parallel-connected tuning element. As an intermediate step towards this goal, we first apply the circuit description to a system composed of an infinite TWTL and a parallel-connected finite TWTL section of length *L* (usually referred to as a ‘stub’^30,31^), as sketched in Fig. 4a. For this geometry one expects reflection at the stub termination and thus resonances building up within the stub. For this study, in order to further corroborate the role played by the phenomenological parameter *κ*, the geometrical cross section of the TWTL is chosen as in Fig. 2a–d, i.e. the individual nanowires have a cross section of 30 × 30 nm^2^ and a gap of 10 nm. Thus, *κ* takes a value of 0.036 and the geometry is expected to show a good agreement with the theory based on Kirchhoff’s laws. Figure 4b shows the simulated near-field intensity distribution within the infinite TWTL when connected in parallel with a high impedance stub (*L* = 120 nm, top) and a low impedance stub (*L* = 180 nm, bottom). The high reflectivity observed for the case of the low impedance stub is a direct consequence of the destructive interference along the output TWTL. Figure 4c shows the reflection amplitude and phase for varying stub length *L* as measured at the stub connection position obtained by full-3D FDTD simulations (red dots). As expected, the reflection amplitude can be tuned over a wide range of values by adjusting *L*. Note that nearly full and nearly zero reflectivity can be obtained. Moreover, the observed modulation period matches *λ*_eff_/2 of the antisymmetric waveguide mode due to the formation of resonances in the stub. When compared to the analytical circuit model (see Supporting Information), the features are qualitatively reproduced fairly well, apart from residual deviations for specific stub lengths, especially around reflection maxima and minima corresponding to low and high impedance stubs, respectively.

**Figure 5:**
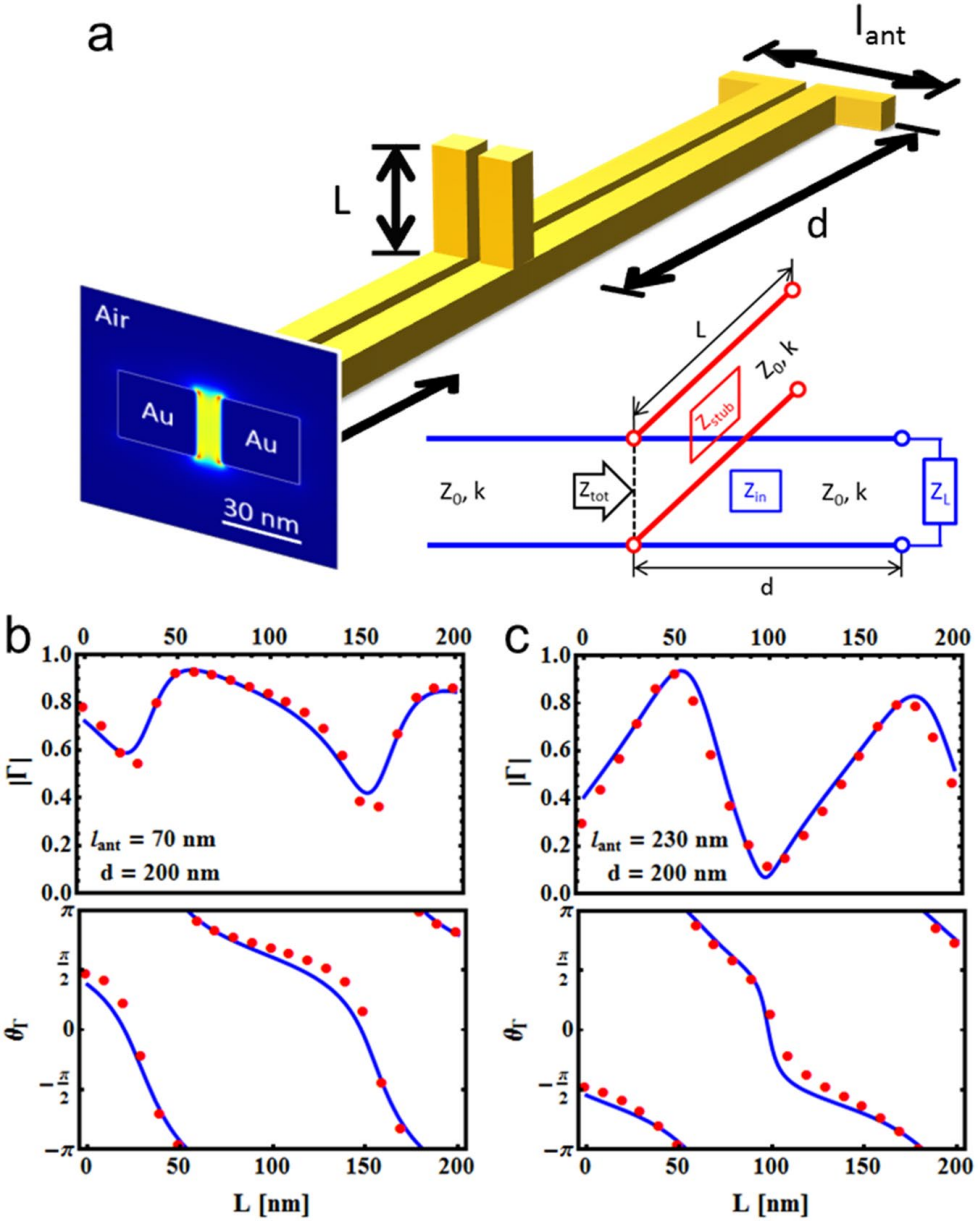
Tuning the reflectivity in a nanoantenna-terminated TWTL by a parallel stub. (**a**) Sketch of the investigated system featuring a finite TWTL terminated by an optical antenna of length *l*_ant_ connected in parallel with a finite stub of length *L* at a distance *d* from the antenna. The shown antisymmetric mode is directly launched from the left and propagates along the nano-sized TWTL. Inset: Equivalent circuit representation of the system. (**b**) Reflection amplitude (top) and phase (bottom) for varying stub length *L* in a system with open end termination (*l*_ant_ = 70 nm) and *d* = 200 nm. (**b**) Reflection amplitude (top) and phase (bottom) for varying stub length *L* in a system with resonant antenna termination (*l*_ant_ = 230 nm) and *d* = 200 nm. The red dots are obtained by fitting of FDTD simulation data with the model described in Eq. 2, while the blue solid lines are obtained by the analytical model (Supporting Information).

Based on the demonstrated ability of tuning the reflectivity by a stub, we now incorporate a parallel stub into a prototypical nanocircuit relevant for applications. In this nanocircuit (shown in Fig. 5a) the concept of impedance matching is applied by connecting a defined stub of length *L* in parallel at a distance *d* from a load nanoantenna of length *l*_ant_ in order to fulfill the condition of minimizing the system’s reflectivity or achieve maximum signal transfer to the nanoantenna. Note that with complex impedances minimum voltage reflectivity and maximum power transfer are two different conditions^18,32^. The equivalent circuit representation is detailed in the inset of Fig. 5a. The complexity of such a system, together with the potentially large scattering background that might be present in the surroundings of the circuit because of the large radiation resistance of the antenna, opens up further relevant issues. In particular, this system is the perfect candidate to check for spurious deviations from the simple theory because of near-field coupling and/or radiative cross-talk between the lumped elements (here, the antenna and the stub) not included in the RF model. An exhaustive study of the practically unlimited number of possible nanocircuits with different combinations of *L*, *d*, and *l*_ant_ is beyond the scope of this work.

Instead, we concentrate on two specific loading conditions, namely an open-end termination (i.e. *l*_ant_ = 70 nm) and a resonant nanoantenna (i.e. *l*_ant_ = 230 nm). As in the classical transmission line theory, the open end termination of the plasmonic TWTL under investigation results in a nearly perfect reflectivity (|Γ| = 97.4%), while the termination with a resonant nanoantenna leads to a minimum in reflectivity (|Γ| = 37.6%) (see Fig. S1, Supporting Information). Figure 5b,c show plots of the simulated reflection amplitude and phase for varying stub length *L* and fixed stub distance *d* = 200 nm (red dots) for open end and resonant nanoantenna termination, respectively. Simulation data for a third, non-resonant antenna of length *l*_ant_ = 290 nm with an intermediate reflection amplitude together with additional distances *d* in the range from 50 nm to 2 μm for each antenna length are presented in Figs S2, S3 and S4 (Supporting Information). When compared to the analytical circuit model (blue solid line, see also Supporting Information) some clear deviations are identified. While this disagreement can again be in part attributed to the small deviations already observed before for the junction of infinite TWTLs, the newly introduced coupling between nearby elements in this system may lead to additional deviations. This might be e.g. due to the radiation resistance of the antenna elements, which results in a large scattering that can be channelled back towards nearby elements (‘photon recycling’)^33^, or due to the coupling between lumped elements, which distorts the local field configuration and thus changes the value of the effective lumped impedance. From all the presented data one can draw some tentative conclusions concerning the degree of agreement between the simulations and the model. Generally, smaller distances *d* between the stub and the load result in larger deviations (see e.g. Fig. S2). On the one side, the fact that the main deviations are observed if *d* gets smaller than about 500 nm is consistent with a mode propagation length of about 900 nm for the guided mode, since coupling effects through guided modes in the antenna-stub cavity become less important as the round trip distance gets longer than the propagation length. On the other side, if scattered fields would be the main source of coupling instead of guided modes, we should see larger disagreement for antenna terminations with large radiation efficiencies, which is not the case. Instead, the system terminated by the resonant load antenna (Figs 5c and S3) seems to show the best agreement, even for a 100 nm distance. Therefore, it is tentatively suggested that the guided fields bouncing back and forth between the stub and the load are responsible for the observed deviations since they result in increased coupling, resulting in larger disagreement for elements that have a high reflectivity for the guided mode such as the open end termination (Figs 5b and S2). Consistently with its intermediate reflectivity value, the non-resonant antenna (*l*_ant_ = 290 nm, Fig. S4) shows a degree of agreement which is in between the open end and the resonant antenna. Lastly, the deviation reaches a maximum when the reflectivity amplitude of the overall system is minimum, meaning that the energy is effectively “stored” in the cavity formed between the stub and the load. For the lumped circuit model to take these effects into account, one should add a dependent current source describing the coupling between the individual elements^10^, with significant additional complexity.

Despite all the mentioned deviations, the lumped circuit model still can serve as a rough guideline to compose complex nano-optical circuitry by taking advantage of the possibility inherent to an analytical model to rapidly scan multi-dimensional parameter spaces for optimization purposes. A limited set of time-consuming numerical simulations can subsequently be performed to verify and fine-tune these pre-optimized parameters to further take into account additional coupling effects. Although in the analysis of experimental data the inaccuracy of the equivalent lumped model may be partly obscured by the resolution and reproducibility of the established nanolithography techniques, such as electron-beam lithography and Ga-based focused ion beam milling, current trends moving e.g. towards single-crystal Au films^34^ and He-based ion beam milling^35,36^ might require the highest level of accuracy in the modelling of plasmonic nanocircuitry. As a final remark, it should be noted that the application of the equivalent impedance approach to plasmonic waveguiding is relatively straightforward only as long as metal-insulator-metal geometries are considered, for which voltages and currents are easily defined. In this respect, however, it should also be mentioned that broader definitions for the impedance of plasmonic waveguides have been proposed in the literature^21^.

## Conclusions

In conclusion, by investigating a junction of plasmonic TWTLs we obtain insight into the reasons for and the strength of the deviations from Kirchhoff’s laws at the junction of plasmonic waveguides. We find that the validity of Kirchhoff’s laws can tentatively be checked via the value of a phenomenological parameter *κ* relating the geometrical parameters of the transmission line with the plasmon effective wavelength. In the regime of small *κ* (which we roughly identify as the values of up to about 0.1) the junction behaves according to Kirchhoff’s theory. Beyond such regime, for large values of the phenomenological parameter, increasing deviations occur and the equivalent impedance description (Kirchhoff’s laws) can only provide rough, but nevertheless useful, guidelines for the design of more complex plasmonic circuitry. Moreover, we investigate the influence of the coupling between individual plasmonic elements with a system composed of a TWTL and a nanoantenna as a load by addition of a suitable stub connected in parallel and give some tentative explanations for the different degrees of agreement observed for various structure parameters.

## Electronic supplementary material

Supporting Information

## References

[1] Novotny L, Hafner C (1994). Light propagation in a cylindrical waveguide with a complex, metallic, dielectric function. Phys. Rev. E.

[2] Takahara J, Yamagishi S, Taki H, Morimoto A, Kobayashi T (1997). Guiding of a one-dimensional optical beam with nanometer diameter. Opt. Lett..

[3] Barnes WL, Dereux A, Ebbesen TW (2003). Surface plasmon subwavelength optics. Nature.

[4] Ozbay E (2006). lasmonics: Merging Photonics and Electronics at Nanoscale Dimensions. Science.

[5] Gramotnev DK, Bozhevolnyi SI (2010). Plasmonics beyond the diffraction limit. Nat. Photon..

[6] Fang Y (2010). Branched Silver Nanowires as Controllable Plasmon Routers. Nano Lett..

[7] Wei H, Wang Z, Tian X, Käll M, Xu H (2011). Cascaded logic gates in nanophotonic plasmon networks. Nat. Commun..

[8] Rewitz C (2014). Coherent Control of Plasmon Propagation in a Nanocircuit. Phys. Rev. Applied.

[9] Bozhevolnyi SI, Volkov VS, Devaux E, Laluet J-Y, Ebbesen TW (2006). Channel plasmon subwavelength waveguide components including interferometers and ring resonators. Nature.

[10] Engheta N, Salandrino A, Alù A (2005). Circuit Elements at Optical Frequencies: Nanoinductors, Nanocapacitors, and Nanoresistors. Phys. Rev. Lett..

[11] Alù A, Engheta N (2008). Tuning the scattering response of optical nanoantennas with nanocircuit loads. Nat. Photon..

[12] Berthelot J (2009). Tuning of an Optical Dimer Nanoantenna by Electrically Controlling Its Load Impedance. Nano Lett..

[13] Greffet J-J, Laroche M, Marquier F (2010). Impedance of a Nanoantenna and a Single Quantum Emitter. Phys. Rev. Lett..

[14] Alù A, Engheta N (2010). Wireless at the Nanoscale: Optical Interconnects using Matched Nanoantennas. Phys. Rev. Lett..

[15] Liu N (2013). Individual Nanoantennas Loaded with Three-Dimensional Optical Nanocircuits. Nano Lett..

[16] Olmon RL, Raschke MB (2012). Antenna–load interactions at optical frequencies: impedance matching to quantum systems. Nanotechnology.

[17] Abasahl B, Santschi C, Martin OJF (2014). Quantitative Extraction of Equivalent Lumped Circuit Elements for Complex Plasmonic Nanostructures. ACS Photonics.

[18] Huang J-S, Feichtner T, Biagioni P, Hecht B (2009). Impedance Matching and Emission Properties of Nanoantennas in an Optical Nanocircuit. Nano Lett..

[19] Li D, Li E-P (2013). Impedance calculation and equivalent circuits for metal–insulator–metal plasmonic waveguide geometries. Opt. Lett..

[20] Alù A, Engheta N (2008). Input Impedance, Nanocircuit Loading, and Radiation Tuning of Optical Nanoantennas. Phys. Rev. Lett..

[21] Kaiser, T., Hasan, S. B., Paul, T., Pertsch, T. & Rockstuhl, C. Impedance generalization for plasmonic waveguides beyond the lumped circuit model. *Phys*. *Rev*. *B* **88** (2013).

[22] Xu Y, Tucker E, Boreman G, Raschke MB, Lail BA (2016). Optical Nanoantenna Input Impedance. ACS Photonics.

[23] Ginzburg P, Orenstein M (2007). Plasmonic transmission lines: from micro to nano scale with *λ*/4 impedance matching. Opt. Express.

[24] Kocabas SE, Veronis G, Miller DAB, Fan S (2008). Transmission Line and Equivalent Circuit Models for Plasmonic Waveguide Components. IEEE J. Sel. Top. Quantum Electron..

[25] Veronis G, Kocabaş ŞE, Miller DAB, Fan S (2009). Modeling of Plasmonic Waveguide Components and Networks. J. Comput. Theor. Nanosci..

[26] Pannipitiya A, Rukhlenko ID, Premaratne M, Hattori HT, Agrawal GP (2010). Improved transmission model for metal-dielectric-metal plasmonic waveguides with stub structure. Opt. Express.

[27] Taminiau TH, Stefani FD, van Hulst NF (2011). Optical Nanorod Antennas Modeled as Cavities for Dipolar Emitters: Evolution of Sub- and Super-Radiant Modes. Nano Lett..

[28] Johnson PB, Christy RW (1972). Optical Constants of the Noble Metals. Phys. Rev. B.

[29] Etchegoin PG, Ru ECL, Meyer M (2006). An analytic model for the optical properties of gold. J. Chem. Phys..

[30] Cheng, D. K. *Field and wave electromagnetics* (Addison-Wesley Publishing Company, 1989).

[31] Reiserer AA, Huang J-S, Hecht B, Brixner T (2010). Subwavelength broadband splitters and switches for femtosecond plasmonic signals. Opt. Express.

[32] Kurokawa K (1965). Power Waves and the ScatteringMatrix. IEEE Trans. Microw. Theory Tech..

[33] Englund D, Fushman I, Vuckovic J (2005). General recipe for designing photonic crystal cavities. Opt. Express.

[34] Huang J-S (2010). Atomically flat single-crystalline gold nanostructures for plasmonic nanocircuitry. Nat. Commun..

[35] Melli M (2013). Reaching the Theoretical Resonance Quality Factor Limit in Coaxial Plasmonic Nanoresonators Fabricated by Helium Ion Lithography. Nano Lett..

[36] Hlawacek, G. & Gölzhäuser, A. *Helium Ion Microscopy* (Springer International Publishing, 2016).

