# Supplementary material for: Limits of Kirchhoff’s Laws in Plasmonics
Source: Sci Rep. 2018 Jan 30;8:1921. doi: 10.1038/s41598-018-20239-x (PMC5789851; doi:10.1038/s41598-018-20239-x)
Supplement: Supplementary file 1 — Supporting Information [file 41598_2018_20239_MOESM1_ESM.pdf]

Supporting Information for manuscript entitled "Limits of Kirchhoff's Laws in Plasmonics"

Gary Razinskas,† Paolo Biagioni,‡ and Bert Hecht*,†,¶

†*Nano-Optics and Biophotonics Group, Department of Experimental Physics 5, University of Würzburg, Am Hubland, D-97074 Würzburg, Germany*

‡*Physics Department, Politecnico di Milano, Piazza Leonardo da Vinci 32, I-20133 Milano, Italy*

¶ *Röntgen Center for Complex Material Systems (RCCM), Am Hubland, D-97074 Würzburg, Germany*

E-mail: hecht@physik.uni-wuerzburg.de

Analytical model for the parallel connection of a stub and an inifinite TWTL

In the following, we adopt well-known formulas from the RF regime, as can be found in standard textbooks,[1] in order to describe plasmonic systems and validate Kirchhoff's analysis at optical frequencies. Let Z_{open} be the impedance of the open-circuit 'load'. A stub with length L can be seen as an open-circuit load attached to a segment of TWTL with length L, therefore its impedance Z_{stub} can be described via

$$Z_{stub} = Z_0 \frac{Z_{open} + Z_0 \tanh(kd)}{Z_0 + Z_{open} \tanh(kd)}. \tag{S1}$$

If this stub is parallel-connected to an infinite TWTL, the total input impedance Z_{tot} measured at the stub connection position is the parallel of Z_{stub} and Z_0, i.e.

$$\frac{1}{Z_{tot}} = \frac{1}{Z_{stub}} + \frac{1}{Z_0}. \tag{S2}$$

Therefore, the reflection coefficient Γ_{tot} at this reference plane can be evaluated as

$$\Gamma_{tot} = \frac{Z_{tot} - Z_0}{Z_{tot} + Z_0} \tag{S3}$$

and is plotted as the blue solid lines in Fig. 4c of the manuscript.

Reflection coefficient of different load antennas

If a TWTL with characteristic impedance Z_0 is loaded by an antenna with impedance Z_L attached to the TWTL termination (as shown in Fig. S1a), the reflection coefficient Γ measured at the load position can be written as

$$\Gamma = \frac{Z_L - Z_0}{Z_L + Z_0}. \tag{S4}$$

Numerically simulated values of Γ obtained by fitting the total intensity I_{total} of the standing wave patterns building up along the input TWTL (Fig. S1b) with the analytical model described by Eq. 2 of the main manuscript are displayed in Fig. S1c for varying antenna length l_{ant}. The reflection amplitude can be strongly controlled by choosing a certain antenna length.

Analytical model for the parallel connection of a stub and a finite TWTL terminated by a load antenna

Knowing the antenna impedance Z_L it is further necessary to derive the input impedance Z_{in} of a system composed of an additional TWTL segment of length d terminated by such antenna. In analogy to Eq. S1, Z_{in} can be calculated as

$$Z_{in} = Z_0 \frac{Z_L + Z_0 \tanh(kd)}{Z_0 + Z_L \tanh(kd)}. \tag{S5}$$

The parallel connection of Z_{in} and Z_{stub} gives the total input impedance Z'_{tot} of the system shown in Fig. 5a, which reads

$$\frac{1}{Z'_{tot}} = \frac{1}{Z_{stub}} + \frac{1}{Z_{in}}. \tag{S6}$$

Again, the reflection coefficient $\Gamma'_{\rm tot}$ measured at the stub connection position can be evaluated as

$$\Gamma'_{tot} = \frac{Z'_{tot} - Z_0}{Z'_{tot} + Z_0} \tag{S7}$$

and is plotted for certain combinations of antenna length $l_{\rm ant}$, stub distance d and length L as the blue solid lines in Figs. 5b-c, S2, S3, and S4.

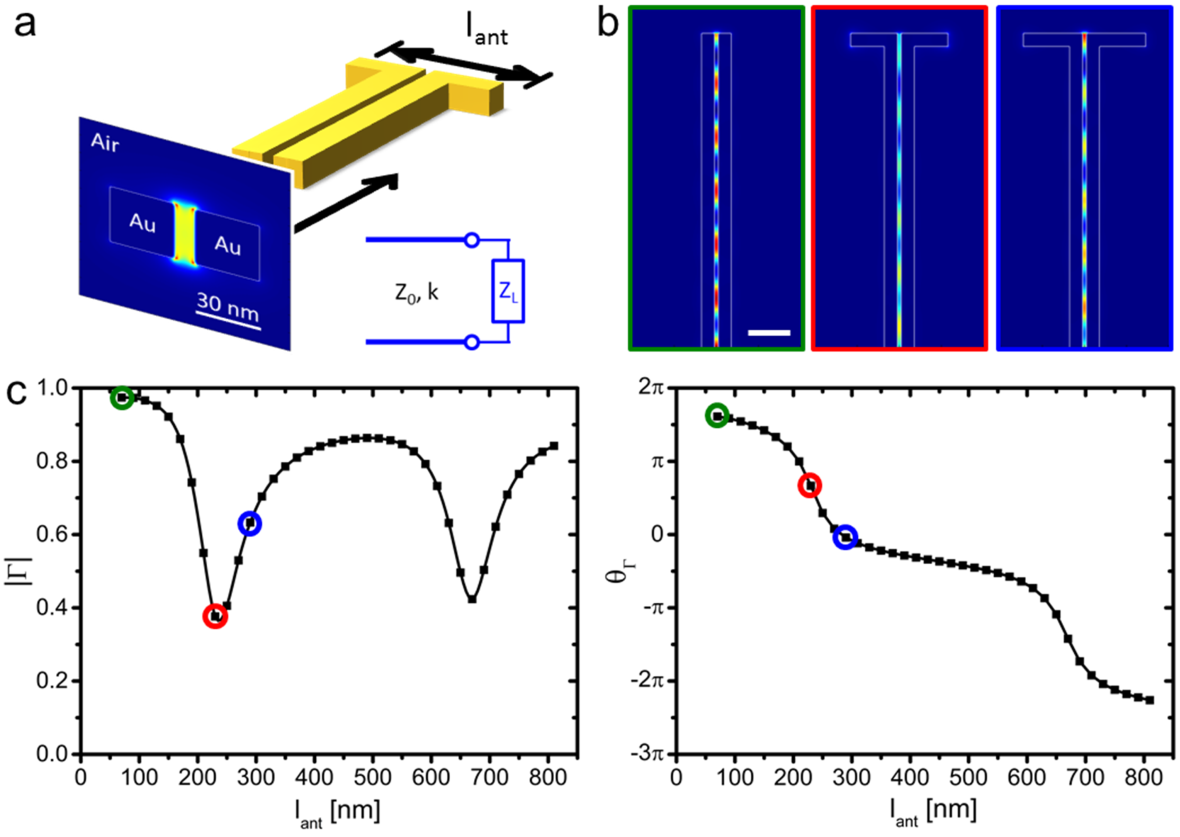

Figure S1: (color online) Tuning the reflectivity by a load antenna's total length. (a) Sketch of the investigated system of a TWTL terminated by an optical antenna of length $l_{\rm ant}$. The shown antisymmetric mode is directly launched from the left and propagates along the nano-sized TWTL. Inset: Equivalent circuit representation of the system. (b) Simulated mode's standing wave pattern along a cut at midheight through the TWTL for an open end termination (left), antenna with $l_{\rm ant} = 230\,\rm nm$ (center), and antenna with $l_{\rm ant} = 290\,\rm nm$ (right). The scale bar in panel b is 100 nm. (c) Reflection amplitude (left) and phase (right) for varying antenna length.

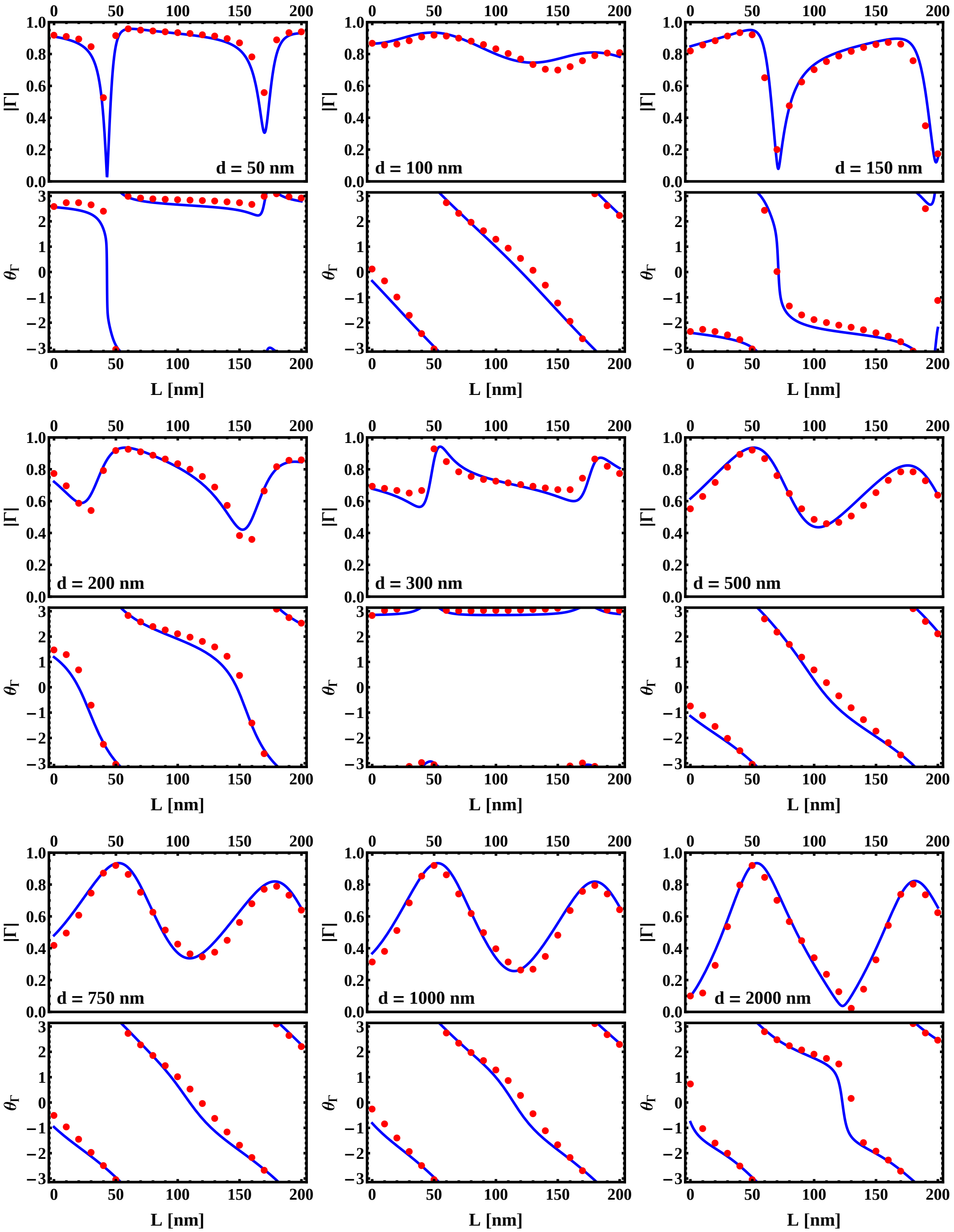

Figure S2: (color online) Reflection amplitude and phase of a system composed of a finite stub of varying length L connected in parallel with an open end terminated TWTL of length $d = 50\,\mathrm{nm}$, $100\,\mathrm{nm}$, $150\,\mathrm{nm}$, $200\,\mathrm{nm}$, $300\,\mathrm{nm}$, $500\,\mathrm{nm}$, $750\,\mathrm{nm}$, $1000\,\mathrm{nm}$, and $2000\,\mathrm{nm}$ (from top left to bottom right). The red dots are obtained by fitting of FDTD simulation data with the model described in Eq. 2 of the main manuscript, while the blue solid lines are obtained by the analytical model.

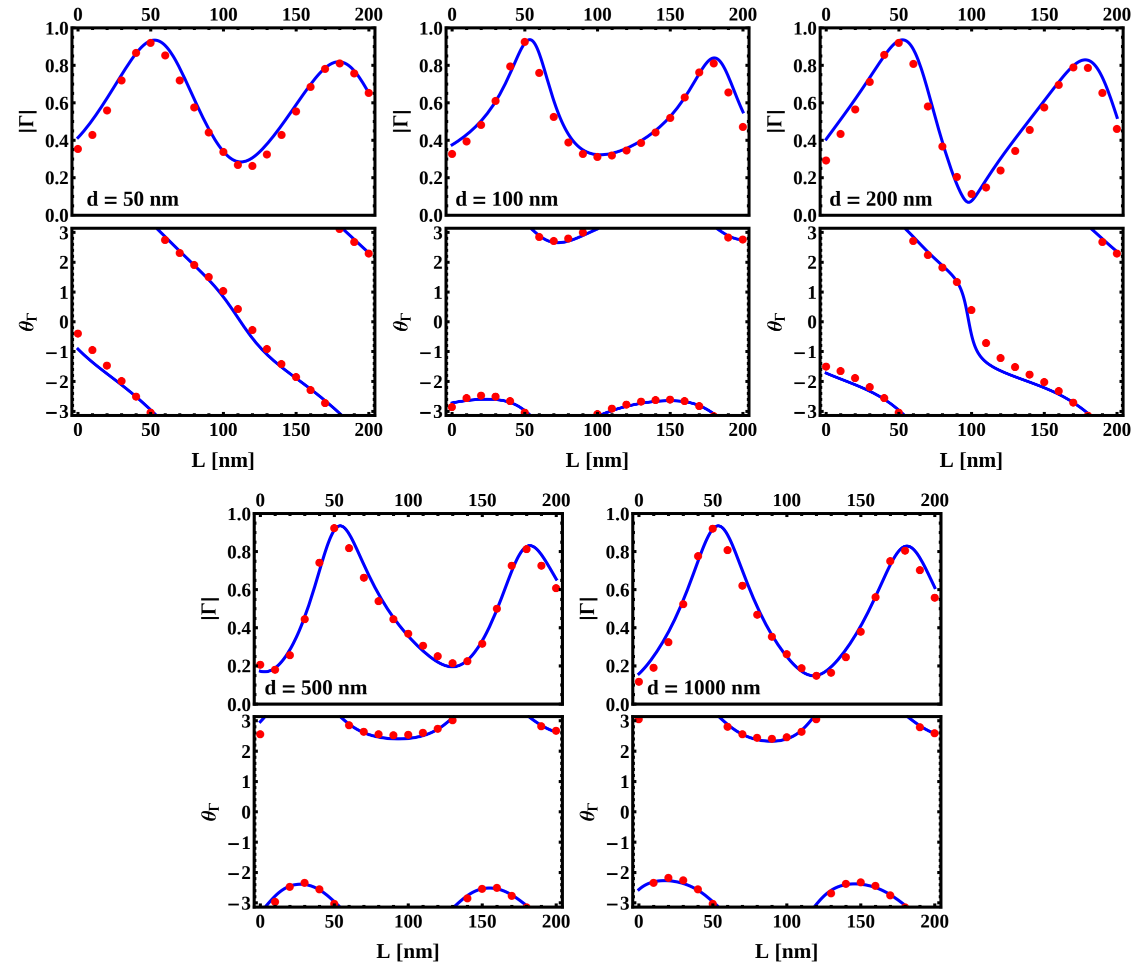

Figure S3: (color online) Reflection amplitude and phase of a system composed of a finite stub of varying length L connected in parallel with a TWTL of length $d = 50\,\mathrm{nm}$, $100\,\mathrm{nm}$, $200\,\mathrm{nm}$, $500\,\mathrm{nm}$, and $1000\,\mathrm{nm}$ (from top left to bottom right) terminated by a resonant antenna of length $l_{\mathrm{ant}} = 230\,\mathrm{nm}$. The red dots are obtained by fitting of FDTD simulation data with the model described in Eq. 2 of the main manuscript, while the blue solid lines are obtained by the analytical model.

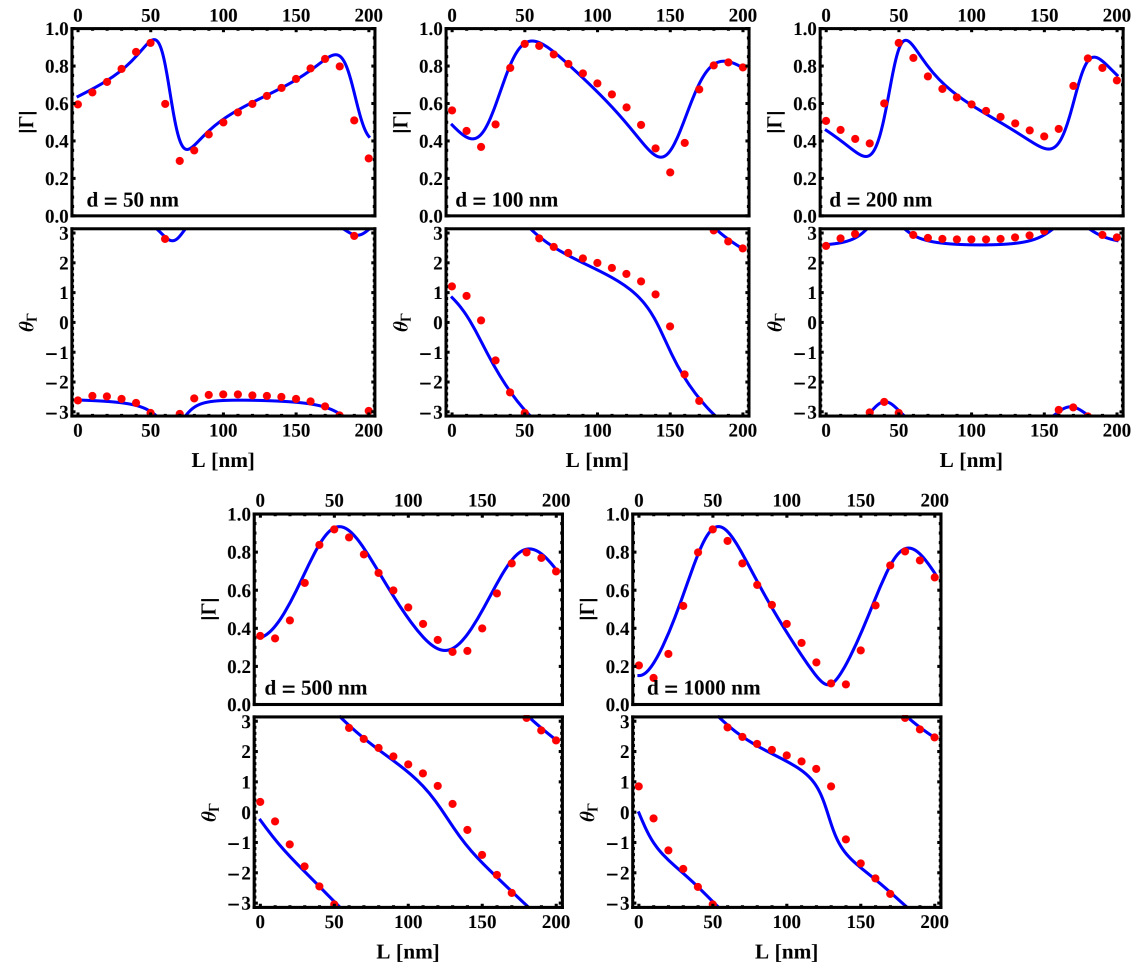

Figure S4: (color online) Reflection amplitude and phase of a system composed of a finite stub of varying length L connected in parallel with a TWTL of length $d = 50\,\text{nm}$, $100\,\text{nm}$, $200\,\text{nm}$, $500\,\text{nm}$, and $1000\,\text{nm}$ (from top left to bottom right) terminated by a resonant antenna of length $l_{\text{ant}} = 290\,\text{nm}$. The red dots are obtained by fitting of FDTD simulation data with the model described in Eq. 2 of the main manuscript, while the blue solid lines are obtained by the analytical model.

References

(1) Cheng, D. K. *Field and wave electromagnetics*; Addison-Wesley Publishing Company, 1989.
